# Local government policy to facilitate healthy and sustainable diets and the broader policy hierarchy: insights from Milan Urban Food Policy Pact cities

**DOI:** 10.1186/s12961-023-00988-6

**Published:** 2023-05-24

**Authors:** Liza R. Barbour, Julie L. Woods, Julie K. Brimblecombe

**Affiliations:** 1 Department of Nutrition, Dietetics & Food, Monash University, Level 1, 264 Ferntree Gully Road, Notting Hill, VIC 3168 Australia; 2 Institute for Physical Activity and Nutrition (IPAN), School of Exercise and Nutrition Sciences, Deakin University, Locked Bag 20000, Geelong, VIC 3220 Australia

**Keywords:** Planetary health, Public policy, Sustainable food production, Sustainable lifestyle, Food systems, Ecological nutrition

## Abstract

**Background:**

Local government authorities are well positioned to change the way food is produced and consumed through the implementation of integrated food policies. By facilitating the uptake of healthy and sustainable diet-related practices, integrated local government food policy can trigger change throughout the food supply chain. This study aimed to provide insights as to how the policy hierarchy surrounding local governments may be influencing local government’s capacity to create integrated food policy.

**Methods:**

Content analysis was conducted on local government food policies (*n* = 36) from signatory cities of the Milan Urban Food Policy Pact were mapped to seven global regions. A set of 13 predetermined healthy and sustainable diet-related practices, organized into three categories: “where to source food”, “what to eat” and “how to eat”, was used to assess the level of integration of each local government food policy. Additional policies from the broader policy hierarchy that were mentioned in each local government food policy were sourced and then screened for relevancy, charted according to their level of administration (local, national, global region, international) and analysed to consider which diet-related practice(s) each broader policy was likely to promote.

**Results:**

Analysis revealed three key insights: (i) local government food policies across all included global regions (*n* = 4) mostly promoted practices in the “where to source food” category, (ii) local government food policies across all global regions referred to policies from higher levels of administration (local, national, global region and international) which tended to also promote practices in the “where to source food” category and (iii) regarding the level of integration, local government food policies in Europe and Central Asia targeted the highest number of diet-related practices.

**Conclusions:**

The level of integration of food policy at national, global region and international levels may be influencing that of local governments. Further research is required to understand why local government food policies are referring to some relevant policies and not others, and to determine whether a greater focus on the diet-related practices of “what to eat” and “how to eat” in policies from higher levels of government would support local governments to also prioritize these practices in their food policies.

**Supplementary Information:**

The online version contains supplementary material available at 10.1186/s12961-023-00988-6.

## Introduction

The global food system is driving diet-related disease and catalysing climate change, prompting calls for a transformation to the way food is both produced and consumed [1–3]. While these challenges are global in nature, it is understood that local government (LG^1^) authorities are well positioned to implement local policy which can contribute to global sustainable development targets [4–8]. The development of LG food policies, “a concerted action on the part of city governments to address food-related challenges”, is an effective approach to improve local food systems and contribute to the broader global transformation [9, p. 9]. LG food policies can take the form of single-issue policy, such as food waste management or urban agriculture strategies [7, 10], or take a more integrated form where the whole food supply chain and different sectors, such as health, agriculture, education, environment, trade, finance, water and waste management, are involved [7, 9, 11–13].

Policy-makers can achieve this integrated policy through three different approaches: (i) creating a new overarching food system policy or plan which brings together activities and interventions from a number of government strategies, (ii) taking a “food in all” approach to ensure food is reflected in policies from other governmental areas and (iii) designing policy interventions which aim to achieve numerous food system outcomes simultaneously [14]. An example of this third type of policy integration is the United Nations’ Decade of Action on Nutrition that encompasses particular policy actions – for example, to create sustainable, resilient food systems for healthy diets, promote social protection and nutrition education, and re-orient trade and investment for improved nutrition – which work together to address multiple food system goals [15]. Common to all three approaches to integrated policy is that they reflect the interconnected nature of the food system by making connections across discrete policy areas, multiple levels of government and between stakeholders from public and private sectors [14]. For this reason, they likely more effectively contribute to food system transformation than single-issue policy approaches [14]. The quest for integrated food policy is ambitious, as policy-makers must balance competing priorities from governmental and private sectors; manage economic, health and environmental trade-offs; and garner political will to buy into such multifaceted interventions [16, 17]. Despite these challenges, a number of LGs globally have developed integrated food policies and set a precedent for others to follow suit.

One approach being taken by LGs globally to address food system issues is to promote population-level dietary change. A healthy and sustainable diet is one with “low environmental impacts which contribute to food and nutrition security and to a healthy life for present and future generations” [18, p. 7]. In a previous study, we reviewed literature published by relevant United Nations agencies or high-level committees to identify 13 desirable healthy and sustainable diet-related practices which are important to achieve such a diet. These practices describe the ways in which individuals can source, store, prepare, consume and dispose of the food that makes up their overall diet to promote human and planetary health, and were categorized to define “where to source food”, “what to eat” and “how to eat” [19] (Table 1). When facilitated together and at a population scale, these 13 practices have the potential to trigger transformative change throughout the food supply chain [14, 19, 20].

**Table 1:** Healthy and sustainable diet-related practices

**Where to source food?**
(1) Select food grown using sustainable food production practices, valuing and respecting Indigenous knowledges (2) Strengthen local food systems by connecting with primary producers (3) Eat seasonally, incorporating native and wild-harvested foods (4) Eat locally available foods
**What to eat?**
(5) Avoid over-consumption beyond caloric requirement (6) Consume no more than recommended animal-derived foods (7) Limit intake of ultra-processed, nutrient-poor and over-packaged food (8) Increase intake of plant-based foods (9) Eat a wide variety of foods to promote biodiversity
**How to eat?**
(10) Adopt food waste minimization strategies (11) Preference homemade meals and share with others (12) Consume safe tap water as preferred drink (13) Breastfeed infants where possible

LGs are promoting these practices in a number of ways, including by exercising legislative and regulatory levers within their power [9, 21]. For example, LGs are updating planning policies to restrict the expansion of fast food restaurants and implementing fiscal policy to incentivize food service and retail businesses to promote healthy and sustainable food options [6, 12, 22, 23]. While they are considered to be well positioned to address food system challenges [24–26], LGs are also beholden to higher levels of government and other agencies when selecting feasible policy actions [5]. Mutually reinforcing policy actions and objectives within and between governments, also referred to as policy coherence [27], can exist vertically among different levels of government, and horizontally across relevant departments and sectors involved in food systems [7, 28, 29]. Efforts to enhance policy coherence within nations and trans-nationally are considered challenging yet critical if the global community is to achieve a more sustainable food system and ultimately meet sustainable development targets [7, 28].

Urban local governments can play a particularly critical role in food system transformation by promoting the population-wide shift to more desirable dietary behaviours, as urban food policies affect large populations [4, 12, 30]. A number of interventions exist to support urban LGs such as the Milan Urban Food Policy Pact (MUFPP^2^), which enables LG authorities in urban settings to publicly commit to creating food systems that are sustainable, inclusive, resilient and safe [13, 25, 27, 31–33]. The MUFPP monitoring framework encourages signatory cities to implement integrated policy interventions which address issues throughout the food supply chain and involve many sectors, therefore promoting horizontal policy cohesion [4]. In 2019, Candel identified that only one quarter of all MUFPP signatory cities had developed an integrated, local food strategy [21]. Similarly, we previously found that only 22% of MUFPP signatory cities’ LG food policies (*n* = 36) identified in a scoping review considered all phases of the food supply chain [34]. Scholars have identified a number of barriers faced by LGs when aiming to develop integrated food policies, such as limited access to funding, “nanny-state” criticisms, industry opposition and pre-emptive laws, where a higher level of government displaces the authority of a lower level of government to take action [5, 6, 9, 10, 30, 35, 36].

This study aims to provide insights as to how the policy hierarchy surrounding LGs may be influencing their capacity to create integrated food policy. This study is particularly concerned with the third type of integrated policy mentioned earlier, where the policy seeks to achieve multiple food systems outcomes simultaneously, and explores this by examining the number of desirable diet-related practices targeted in each LF food policy. To do this, a secondary analysis of the LG food policies identified through our previous scoping review was conducted to answer two key research questions: (i) Which diet-related practices are most frequently targeted by LG food policies in each global region? (ii) Which related policies from local, national, global region and international levels of administration are referred to by LGs in their food policies, and which diet-related practices do they target?

## Methods

This study analysed 36 LG food policies identified in a previously published scoping review [34]. In brief, this scoping review involved an initial search of key terms “local government”, “policy”, “food” and “environmental sustainability” with each of their synonyms across five databases: Scopus, Medline, CINAHL Plus, Global Health and Pro-quest – Agricultural & Environmental Science Collection. A total of 2624 peer-reviewed studies were identified. Titles and abstracts were double screened and eligible full-text papers were retrieved and further screened by two researchers, as guided by the Preferred Reporting Items for Systematic Reviews and Meta-Analyses Extension for Scoping Reviews (PRISMA-ScR) checklist [37] (Additional file 1: A: PRISMA flowchart, B: Inclusion criteria). Studies were included if they were published after 2015 and described one or more LG food policies implemented within an urban setting, specifically a signatory city of the MUFPP (*n* = 199). MUFPP signatory cities were deemed a useful sample for this study as they demonstrate a commitment to food system transformation using the pre-eminent international urban food policy framework for LGs. They also represent a diversity of global regions and therefore provide insight into LG food policy practices across the globe. A final 27 studies met the inclusion criteria which were further screened to identify citations to 36 eligible LG food policies. These 36 policy documents were retrieved from the grey literature and data charted in an excel document including the name of the LG food policy, name of MUFPP signatory city(ies), geographic location of LG jurisdiction according to the United Nations’ seven global regions [38], a description of policy actions, the healthy and sustainable diet-related practice(s) [19] targeted by policy actions (as presented in Table 1) and names of broader policies referred to within the LG food policy.

For the current study, charted data were further analysed to explore how the policy hierarchy surrounding LGs may influence the level of integration in LG food policy, by examining which diet-related practices were most frequently targeted by LG food policies in each global region. First, the name of each LG food policy (*n* = 36) and its corresponding MUFPP signatory city, country and global region within which it was developed, and the healthy and sustainable diet-related practice(s) targeted by the policy action, were tabled (Additional file 1: C). Second, for each diet-related practice, the proportion of LG policies in each global region targeting the diet-related practice was determined. The number of diet-related practices targeted in each LG food policy was also tabulated and presented as a percentage of the total number (*n* = 13). The overall average percentage for each global region was calculated. To examine the policy hierarchy, the names of policies referred to within each LG food policy were screened for relevance. A policy was considered relevant if the title suggested it related to one or more of the six phases of the food supply chain (agricultural production, distribution, processing, food retail, consumption and waste [39]) and/or addressed one or more challenges created by the food system such as food insecurity. Relevant policy documents were then sourced from the grey literature, and the title and preamble were examined by the first author to determine (i) the level of policy administration: local, national, global region and international (Table 2), and (ii) the category(ies) of diet-related practices most prominently targeted (Additional file 1: D).

**Table 2:** Level of policy administration and description

Level of administration	Description
Local	Policies administered by local, state and provincial authorities as well as those spanning multiple local government areas
National	Policies administered by national/federal levels of government
Global region	Policies administered across multiple countries within each of the seven global regions, as defined by the United Nations [38]
International	Policies administered at a global level, involving one or more global regions, including incentive policies and networks

## Results

As previously reported [34], of the 36 LG food policies included in this analysis, most were from the global regions of Europe and Central Asia (*n* = 19, 53%) and North America (*n* = 10, 28%). No policies from three global regions (East Asia and Pacific, Middle East and North Africa, or South Asia) met the inclusion criteria.

### Diet-related practices targeted by LG food policies in each global region

**Fig. 1:**
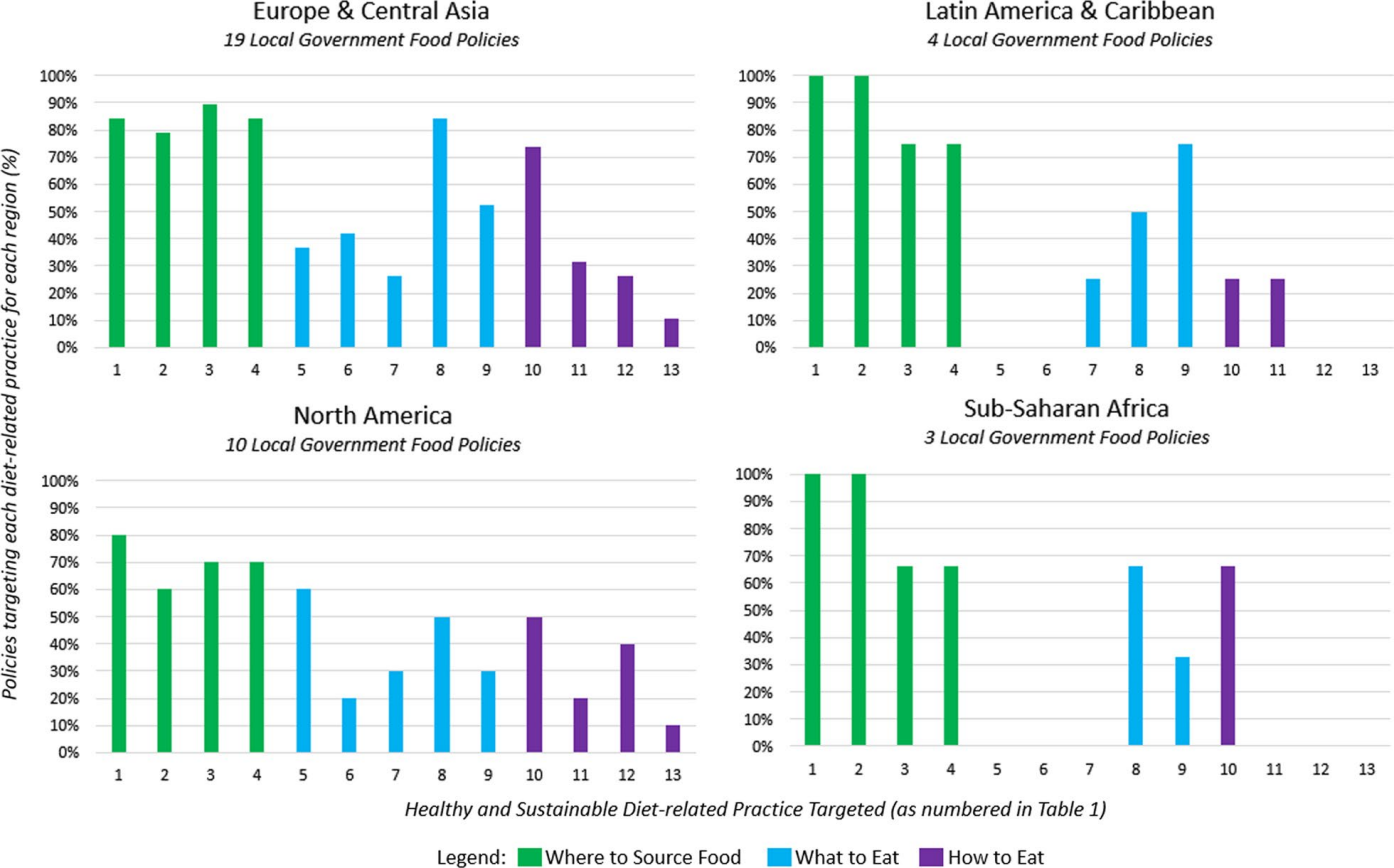
Percentage of policies targeting diet-related practice, by global region

As shown in Fig. 1, the diet-related practices most consistently targeted across the four included global regions were from the “where to source food” category (green; practices 1–4, as listed in Table 1). All LG food policies from Latin America and Caribbean and sub-Saharan Africa encouraged citizens to select food grown using sustainable production practices (practice 1) and strengthen local food systems by connecting with primary producers (practice 2).

Practices concerned with “what food is eaten” (blue; practices 5–9) were less consistently targeted in LG food policies across all regions. Policy aiming to increase the intake of plant-based foods (practice 8), which included efforts made to grow and harvest fruit and vegetables within the local area, was promoted in over half of all LG food policies for each global region. However, less than half of all LG food policies for each global region targeted practices 6 and 7. Consuming no more than recommended amounts of animal-derived foods (practice 6) was targeted by only 42% of LG food policies in Europe and Central Asia, 20% in North America and in no LG food policies in Latin America and the Caribbean or sub-Saharan Africa regions.

Of the practices in the “how food is eaten” category (purple; practices 10–13) waste minimization strategies (practice 10) were most consistently targeted across all regions. The promotion of breastfeeding was included in only 11% of LG food policies from Europe and Central Asia, 10% from North America and none from the other two included global regions.

In terms of the integrated nature of LG food policies analysed, there was at least one policy in each of the four global regions which targeted a diet-related practice from each of the three categories (shown as green, blue and purple) (Additional file 1: C). This demonstrates the third type of policy integration as described in the introduction, whereby the LG food policy aims to achieve multiple food systems outcomes simultaneously. Only two LG food policies targeted all 13 diet-related practices, one from Europe and Central Asia (the London Food Strategy) and one from North America (the Toronto Food Strategy). According to an average of the percentage of diet-related practices targeted in policies for each region, LG policies in Europe and Central Asia targeted the highest number of diet-related practices (53%), followed by North America (45%), Latin America and Caribbean (42%), and then sub-Saharan Africa (38%). However, the latter two regions also had the least number of LG food policies included in the analysis (*n* = 4 and *n* = 3, respectively).

### Local, national, global region and international policies referred to within LGs food policies

LG food policies referred to a range of relevant policies implemented at local, national, global region and international levels (Table 3). These policies represent various types of policy, including legislation (e.g. United States Farm Bill), governing frameworks (e.g. the Sustainable Development Goals) and guiding documents (e.g. International Panel of Experts on Sustainable Food Systems or “IPES^3^” Food Common Food Policy).

**Table 3:** Policies referred to within LG food policies, by level of administration (local, national, global region or international – as defined in Table 2)

Level of policy administration	Global regions			
	Europe and Central Asia	North America	Latin America and Caribbean	Sub-Saharan Africa
Local	“Superb Food” (sustainable urban and peri-urban food provision) in Zurich, Rotterdam and their surrounding local governments^A,B^, Zurich Cultural Land Initiative (Switzerland)^A^, Quality Charter for Agriculture and Food Economy (Switzerland)^A,B^, Healthy Schools London^A,B,C^, London Healthy Workplace Charter^A,B,C^	Provincial Local Food Act 2013 (Toronto and surrounding municipalities)^A,B^, Regional Growth Strategy (Vancouver)^A^, Affordable Housing Strategy (Vancouver)^A^, British Columbia’s Climate Action Plan (Vancouver)^A,B,C^, Mandatory Recycling and Composting Ordinance 2009 (San Francisco)^C^	Municipal Secretariat of Supply (Belo Horizonte)^A^, City Region Food System of Medellin (31 municipalities surrounding Medellin)^A,B,C^, Plan of Development for the Metropolitan District of Quito of 2015 (Ecuador)^A^	Economic Development Strategy (Cape Town)^A^, Poverty Alleviation Strategy (Cape Town)^A^
National	Federal Trans Fatty Acid regulation (Austria)^B^, Animal Welfare Act (Austria)^A,B^, Federal Procurement Act (Austria)^A,B^, National legislation to introduce organic procurement in school canteens (Italy)^A,B^, National Health Service’s Public Health Directorate (United Kingdom)^B^, National School Food Plan 2013 (United Kingdom)^A,B,C^	Love Food Hate Waste Canada (Canada)^A,B,C^, Farm Bill legislation (United States)^A,B,C^, Supplemental Nutrition Assistance Program (United States)^A,B,C^, Child Nutrition Act (United States)^A,B,C^, WIC program (United States)^A,B,C^, SNAP Education (United States)^A,B,C^	Family Farming Law (Brazil)^A,B,C^, Plan for Food and Nutrition Security 2016–2028 (Columbia)^A,B^, Organic Agriculture Law of 2007 (Ecuador)^A,B^, Food Sovereignty Law of 2009 (Ecuador)^A,B,C^, National Plan for Good Living of 2013 (Ecuador)^A,B,C^	National Agriculture, Fisheries & Food Act 2013 (Nairobi)^A,B^, Crops Act 2013 (Nairobi) ^A^
Global region	Smart Food Cities for Development project (EU)^A,B, C^, IPES Food’s “Towards a Common Food Policy for the European Union” (EU)^A,B,C^, European Parliament resolution on Fair Trade and Development (EU)^A^, Edible Cities Network (EU)^A,B,C^, Food labelling and Country of Origin legislation (EU)^A,B^	Nil referred to	Nil referred to	Harare Declaration on Urban and Peri-urban Agriculture 2003 (Kenya, Malawi, Swaziland, Tanzania and Zimbabwe)^A,B^
International	Sustainable Development Goals^A,B,C^, Paris Agreement, Milan Urban Food Policy Pact^A,B,C^, United Nations Decade of Action on Nutrition^A,B,C^, “Sustainable Schools” initiative^A,B,C^, C40 Cities Advancing Towards Zero Waste Declaration^A,B,C^			

Targeted diet-related practices from the three categories; ^A^ “Where to source food” (practices 1–4), ^B^ “What to eat” (practices 5–9) and ^C^ “How to eat” (practices 10–13)

Across all global regions, most policies targeted diet-related practices in the “where to source food” category (see legend in Table 3). For example, at the global region level: Europe’s “Edible Cities Network” and “Food Labelling and Country of Origin” legislation and Africa’s “Harare Declaration on Urban and Peri-Urban Agriculture”; at the national level, the United States “Farm Bill” legislation, Italy’s legislation to introduce organic procurement in school canteens and Brazil’s “Family Farming Law”; and at the local level, Zurich’s cultural land initiative, Vancouver’s “Regional Growth Strategy” and Belo Horizonte’s “Municipal Secretariat of Supply”. Fewer policies targeted diet-related practices in the “what to eat” and “how to consume” food categories, for example, Austria’s “Trans Fatty Acid” regulation and Canada’s “Love Food, Hate Waste” campaign. Some policies demonstrate policy integration by targeting diet-related practices from all three categories, including three of the five policies administered at a global region level for Europe and Central Asia and all the five policies implemented at the international level.

## Discussion

This research analysed 36 LG food policies from MUFPP signatory cities to provide insights as to how the policy hierarchy surrounding local governments may be influencing LG’s capacity to create integrated food policy which targets all desirable diet-related practices. Analysis revealed three key insights: (i) LG food policies across all included global regions (*n* = 4) mostly promoted practices in the “where to source food” category, and targeted practices related to “what to eat” and “how to eat” less; (ii) LG food policies across all global regions referred to policies from higher levels of administration (local, national, global region and international) which tended to also promote practices in the “where to source food” category; and (iii) regarding the level of integration, LG food policies in Europe and Central Asia targeted the highest number of diet-related practices.

This study adds to our previous findings by highlighting that LG food policies in all included global regions are prioritizing policy actions in the “where to source food” category. These policy actions encourage consumers to select food grown using sustainable food production practices, connect with primary producers, and eat seasonally and locally available foods [34]. Candel’s (2019) review of LG food policies from MUFPP signatory cities also identified that local food production and improving agricultural practices were among the most common policy goals [21].

This analysis suggests that practices concerned with “what to eat” and “how to eat” may require greater attention, with the exception of strategies to minimize food waste which are currently being prioritized in all regions. This may be explained by considering the traditional and well-established roles of LGs. Known to focus on “roads, rates and rubbish”, LGs also possess expertise and authority in land use management and collaboration with primary food producers. These responsibilities are likely to favour policy to address the way food is produced, sourced by consumers and disposed of, as reflected in our findings [5, 6, 10]. Interestingly, the global region with LG food policies targeting the most diet-related practices was Europe and Central Asia, home to Milan’s integrated food policy which is recognized as best practice globally [40]. This region is also home to a number of integrated policies at the global region level such as IPES Food’s “Towards a Common Food Policy for the European Union”, which encourages the development of integrated food policy at all levels in the policy hierarchy [20].

Policies implemented at higher levels of government are likely to play a role in dictating which policy actions are feasible for LGs, and ultimately which diet-related practices are prioritized. For example, Brazil’s “Family Farming Law” enforces that LGs spend at least 30% of their food procurement budget to buy food directly from local family farms for publicly funded facilities, promoting practices in the “where to source food” category. This national legislation is implemented at the local level and has strengthened links between primary producers and LG policy-makers. It has financially benefited the local food system and has been demonstrated to benefit consumers, for example, by increasing the nutritional quality of school menus [41]. The present study found that most LG policies from Latin America and the Caribbean are promoting sustainable food production practices, connection with primary producers and consumption of seasonally and locally available foods, all part of the “where to source food” category of diet-related practices. A similar example of higher-level policy that is likely influencing the feasibility of LG policy actions in the United States is the United States “Farm Bill” referred to in Chicago’s “Go to 2040 Regional Comprehensive Plan”. This policy mandates actions across each level of government to improve local food production and consumer access to local food, for example, through an investment in farmers markets at the LG level. While this policy has addressed various food system issues such as emergency food relief, nutrition and food marketing, debates for a more integrated national food policy are ongoing in an effort to equally address other food system challenges [16]. These two examples highlight the levers available to LGs when supportive national policy exists, and imply that policy coherence from higher levels of government may support LGs to prioritize diet-related practices from all three categories.

This study suggests that in light of a growing urgency to improve human and planetary health, LG policy-makers require greater support to target diet-related practices which will have the greatest impact. While some LG authorities have demonstrated their ability to develop integrated policies which promote all diet-related practices (for example, the London Food Strategy [42] and Toronto’s Food Strategy), they remain rare. As proposed by Parsons (2019), having dedicated food system policies like these is one of three approaches to achieving integrated food policy [14]. Other approaches, such as taking a “food in all” approach to address food system challenges across multiple policies are also believed to be effective provided they consider the interconnected nature of food systems. Springmann et al. (2020) demonstrated that practices with the greatest potential to promote human and planetary health are those which limit animal-derived foods, in particular beef and dairy, increase wholegrain and plant-based foods, and avoid over-consumption [43], which all sit within the “what to eat” category of desirable diet-related practices. This study identified that no national-level dietary guideline policies which can guide “what to eat” were referred to in the 36 LG food policies analysed, which raises questions regarding why this might be the case. Implementation of more progressive national dietary guidelines, as recommended by the WHO and the Food and Agriculture Organization in *Sustainable healthy diets—Guiding principles* (2019), may support LGs to target these “what to eat” diet-related practices provided they are part of an integrated policy approach [11, 14].

### Limitations and further research

The data analysed comes from a subset of policy documents identified through a previously conducted scoping review, where only signatory cities to the MUFPP were included and policy actions from all global regions were not represented. Further research could examine the direct policy hierarchy surrounding each LG food policy to extend the knowledge gained from our analysis which grouped all referenced policies by administration level. Further research could also compare LG food policy-making across low-, middle- and high-income country contexts, and peri-urban, regional and rural areas, to identify where inequities may exist. It was outside the purpose of this study to examine the nature of influence that relevant policies referred to in LG food policies had on policy-making processes, for example- whether they were a source of funding or a regulatory standard. Further research is required to explore the nature and level of influence of these vertical relationships with regard to healthy and sustainable diets, to ensure future research and practice is focused on those with the greatest level of influence. The authors recognize that the reasons why policy-makers refer to another policy are varied; therefore, caution is required when making assumptions about why one policy is mentioned and another excluded. It was also beyond the scope of this study to determine whether policies targeted the desirable diet-related practices in their entirety. For example, policy actions promoting the first practice to “select food grown using sustainable food production practices, valuing and respecting Indigenous knowledges” were counted as targeting this practice, regardless of whether they referred to actions to value and respect Indigenous knowledges.

## Conclusions

Local government authorities are well positioned to change the way food is produced and consumed, through the implementation of integrated food policies. However, of the 36 local government food policies from Milan Urban Food Policy Pact signatory cities examined, integrated food policies which target all desired diet-related practices and phases of the food supply chain were rare. There is a need for further research to understand if and how networks such as the MUFPP impact the capacity of LGs to take a holistic approach in developing integrated food policies and, more specifically, the way they prioritize diet-related practices during the policy-making process. This study also showed that the level of integration of food policy at national, global region and international levels may be influencing that of LGs, as policies administered at higher levels within the policy hierarchy favoured policy actions related to where consumers source their food from, which strengthen local food production and shorten food supply chains. To trigger the broader food system transformation that is required, consumers must also change the types of food they are eating and how they are eating them, in particular eating less animal-derived and ultra-processed foods, more wholegrain and plant-based foods, and avoiding the over-consumption of food in general. Further research is required to understand why local governments seem to be prioritizing policy which improves the way food is produced and sourced by consumers, and to determine whether a greater focus on “what to eat” and “how to eat” in policies from higher levels of government would support local government to also prioritize these practices in their food policies.

## Supplementary Information

**Additional file 1: A.** PRISMA-ScR Flowchart of screening for included studies in published scoping review. **B.** Inclusion and exclusion criteria for initial search strategy in published scoping review. **C.** Proportion of diet-related practices targeted in LG food policies, organised by geographic location of the administering LG authority (Global Region, Country and City). **D.** Names of relevant policies referred to within each LG food policy, organised by global region, country and signatory city.

## Data Availability

All data reported in this study is included in previous articles published by the same authors (citations 19 and 34), this published article and/or its supplementary information.

## Footnotes

1 LG: Local Government.

2 MUFPP: Milan Urban Food Policy Pact.

3 IPES: International Panel of Experts on Sustainable Food Systems.
