# Supplementary material for: Local government policy to facilitate healthy and sustainable diets and the broader policy hierarchy: insights from Milan Urban Food Policy Pact cities
Source: Health Res Policy Syst. 2023 May 24;21:35. doi: 10.1186/s12961-023-00988-6 (PMC10207704; doi:10.1186/s12961-023-00988-6)
Supplement: Supplementary file 1 — Additional file 1: A. PRISMA-ScR Flowchart of screening for included studies in published scoping review. B. Inclusion and exclusion criteria for initial search strategy in published scoping review. C. Proportion of diet-related practices targeted in LG food policies, organised by geographic location of the administering LG authority (Global Region, Country and City). D. Names of relevant policies referred to within each LG food policy, organised by global region, country and signatory city. [file 12961_2023_988_MOESM1_ESM.docx]

**Supplementary Material A**

PRISMA -ScR Flowchart^[[1]](#footnote-1)^ of screening process for included studies in published scoping review^[[2]](#footnote-2)^

Records identified through database searching
(n = 2624)

IDENTIFICATION

Duplicates removed
(n = 921)

Records screened for title and abstract (n = 1703)

Records excluded
(n = 1556)

SCREENING

Full text articles screened for eligibility
(n = 147)

Reasons for exclusion (n = 102) as defined in Supplementary Material B

Policy (n = 31)

Outcome (n = 16)

Local Government Involvement (n = 15)

Settings (n = 15)

Study (n = 10)

Cannot access (n = 11)

Language (n = 4)

ELIGIBILITY

Studies excluded during data extraction process (n = 18)

INCLUDED

Final included studies
(n = 27)

**Supplementary Material B**

Inclusion and exclusion criteria for initial search strategy in published scoping review^[[3]](#footnote-3)^

| *Criterion* | *Definition* |
| --- | --- |
| Policy | Policy included any plan, action, intervention, initiative, activity or strategy which had pre-determined intentions (goals, objectives, targets) accompanied by a planned approach or work plan to achieve the desired outcome. Ad hoc activities were not included unless they were part of a policy. Policies could be documented in many forms such as regulatory or non-regulatory statements, websites and strategic reports. Hypothetical scenarios such as simulation or modelling were not included. Food Policy Councils were included, provided they were initiated by local government (or have significant involvement). |
| Outcome | The intended outcome of the policy must have included the promotion of at least one healthy and sustainable diet-related practice, as outlined in Table 1. The targeted diet-related practice(s) must have been clearly stated. The policy must have been designed with consideration of environmental sustainability therefore policies aiming to address overweight, obesity, food insecurity, veganism, vegetarianism or cancer were not included unless environmental sustainability outcomes were considered explicitly. Policies promoting urban agriculture, food safety and sustainable farming were not included unless the desired diet-related practice was considered explicitly. Urban agriculture policies which described the intention to increase dietary consumption of locally grown, seasonal and/or plant-based foods were eligible for inclusion. |
| Local Government Involvement | The policy must have been implemented at a local government level and involve local government employees as stakeholders. Involvement could range from lead implementer, funding provision or consultation representative. The terminology used for local government varies and included; county, municipality, local government area, province, shire, region, council, office. |
| Settings | The policy must have been implemented in an urban setting, specifically in one of the MUFPP signatory cities (n = 199 as documented on 22 July 2019). |
| Study | The publication must have been available in English, published in or after 2015, include adequate detail to discern relevance. Any study type – reviews, dissertations, conference proceedings, etc – was considered. |

**Supplementary Material C**

Proportion of diet-related practices targeted in Local Government (LG) food policies, organised by geographic location of the administering LG authority (Global Region, Country and City)

| *Local Government Food Policy* | | | | | *Healthy and Sustainable Diet-Related Practice^*^* | | | | | | | | | | | | | *Diet-related practice targeted*  *n (%)* |
| --- | --- | --- | --- | --- | --- | --- | --- | --- | --- | --- | --- | --- | --- | --- | --- | --- | --- | --- |
| *Global Region* | *Country* | *Signatory City (MUFPP)* | *LG Food Policy Name* | *1* | | *2* | *3* | *4* | *5* | *6* | *7* | *8* | *9* | *10* | *11* | *12* | *13* |  |
| **Europe & Central Asia**  *(19 LG Food Policies)* | Austria | Vienna | OkoKauf Wien ('EcoBuy') Green Public Procurement program (2012) |  | |  |  |  |  |  |  |  |  |  |  |  |  | 5 (38%) |
|  | Belgium | Gent | Gent Local Food Policy "Ghent en Garde" (2016) |  | |  |  |  |  |  |  |  |  |  |  |  |  | 7 (54%) |
|  | Denmark | Copenhagen | Organic Programme - House of Food (2013) |  | |  |  |  |  |  |  |  |  |  |  |  |  | 11 (74%) |
|  | France | Paris | Topager "WildRoof" (2013) |  | |  |  |  |  |  |  |  |  |  |  |  |  | 7 (54%) |
|  |  |  | Paris Strategy for Sustainable Food (2018) |  | |  |  |  |  |  |  |  |  |  |  |  |  | 11 (74%) |
|  |  |  | Main Verte ‘Green Hand’ Charter (2003) |  | |  |  |  |  |  |  |  |  |  |  |  |  | 6 (46%) |
|  | Italy | Multiple | National Guidelines for School Catering (2010) Ancona, Bari, Bologna, Cagliari, Milan, Rome |  | |  |  |  |  |  |  |  |  |  |  |  |  | 9 (69%) |
|  |  | Bologna | Community rooftop garden of Via Gandusio (2011) |  | |  |  |  |  |  |  |  |  |  |  |  |  | 5 (38%) |
|  |  | Catania | ‘Orti e arte’ - Vegetable Gardens and Arts (2018) |  | |  |  |  |  |  |  |  |  |  |  |  |  | 4 (31%) |
|  |  | Rome | Sustainable Food Procurement for Schools in Rome (2013) |  | |  |  |  |  |  |  |  |  |  |  |  |  | 8 (62%) |
|  |  | Turin | City of Turin: Guidelines on School Canteen Services (2015) |  | |  |  |  |  |  |  |  |  |  |  |  |  | 11 (74%) |
|  | Netherlands | Rotterdam | Edible City Solution initiatives (2018) |  | |  |  |  |  |  |  |  |  |  |  |  |  | 5 (38%) |
|  | Spain | Barcelona | Seasonal food for kindergartens (2013) |  | |  |  |  |  |  |  |  |  |  |  |  |  | 7 (54%) |
|  |  |  | Barcelona Green Infrastructure and Biodiversity Plan (2020) |  | |  |  |  |  |  |  |  |  |  |  |  |  | 2 (15%) |
|  | Switzerland | Zurich | Sustainable Food Procurement Regulations in Zurich City (2010) |  | |  |  |  |  |  |  |  |  |  |  |  |  | 3 (23%) |
|  | UK | Bristol | Bristol's Good Food Action Plan (2015) |  | |  |  |  |  |  |  |  |  |  |  |  |  | 5 (38%) |
|  |  | Brighton & Hove | Spade to Spoon: Digging Deeper. A food strategy and action plan for Brighton and Hove (2012) |  | |  |  |  |  |  |  |  |  |  |  |  |  | 12 (92%) |
|  |  | London | Flagship Food Boroughs in London (2014) |  | |  |  |  |  |  |  |  |  |  |  |  |  | 6 (46%) |
|  |  |  | The London Food Strategy (2018) |  | |  |  |  |  |  |  |  |  |  |  |  |  | 13 (100%) |
|  | ***Total number of policies at regional level targeting each diet-related practice*** | | | 16 | | 15 | 17 | 16 | 7 | 8 | 5 | 16 | 10 | 14 | 6 | 5 | 2 | Average 53% |
|  | ***Proportion (%) of policies targeting each diet-related practice by total number of LG food policies for region (n 19)*** | | | 84 | | 78 | 90 | 84 | 37 | 42 | 26 | 84 | 53 | 74 | 46 | 26 | 11 |  |

| *Local Government Food Policy* | | | | *Healthy and Sustainable Diet-Related Practice^*^* | | | | | | | | | | | | | | | | | | *Diet-related practice targeted*  *n (%)* |
| --- | --- | --- | --- | --- | --- | --- | --- | --- | --- | --- | --- | --- | --- | --- | --- | --- | --- | --- | --- | --- | --- | --- |
| *Global Region* | *Country* | *Signatory City (MUFPP)* | *LG Food Policy Name* | *1* | *2* | *3* | *4* | *5* | *6* | | *7* | *8* | | *9* | *10* | | *11* | | *12* | | *13* |  |
| **Latin America & Caribbean**  *(4 LG Food Policies)* | Brazil | Multiple | National School Feeding Program of Brazil - Sustainable Schools initiative (2009) Araraquara, Belo Horizonte, Curitiba, Porto Alegre, Praia, Rio de Janeiro, Sao Paulo |  |  |  |  |  | |  |  |  | |  |  | |  | |  | |  | 6 (46%) |
|  |  | Belo Horizonte | Belo Horizonte Food Security Policy (2011) |  |  |  |  |  | |  |  |  | |  |  | |  | |  | |  | 4 (31%) |
|  | Columbia | Medellin | Public Policy on Food Security, Food Sovereignty and Nutrition (2005) |  |  |  |  |  | |  |  |  | |  |  | |  | |  | |  | 6 (46%) |
|  | Ecuador | Quito | Participatory Urban Agriculture Project (AGRUPAR) (2000) |  |  |  |  |  | |  |  |  | |  |  | |  | |  | |  | 6 (46%) |
|  | ***Total number of policies at regional level targeting each diet-related practice*** | | | 4 | 4 | 3 | 3 | 0 | | 0 | 1 | | 2 | 3 | 1 | 1 | | 0 | | 0 | | Average 42% |
|  | ***Proportion (%) of policies targeting each diet-related practice by total number of LG food policies for region (n 4)*** | | | 100 | 100 | 75 | 75 | 0 | 0 | | 25 | 50 | | 75 | 25 | | 25 | | 0 | | 0 |  |
| **North America**  *(10 LG Food Policies)* | Canada | Toronto | Toronto Food Strategy (2010) |  |  |  |  |  |  | |  |  | |  |  | |  | |  | |  | 13 (100%) |
|  |  |  | Toronto Golden Horseshoe Food and Farming Action Plan (2012) |  |  |  |  |  |  | |  |  | |  |  | |  | |  | |  | 4 (31%) |
|  |  |  | Toronto's Long-Term Waste Management Strategy (2016) |  |  |  |  |  |  | |  |  | |  |  | |  | |  | |  | 1 (8%) |
|  |  | Vancouver | Regional Food System Action Plan Metro Vancouver (2016) |  |  |  |  |  |  | |  |  | |  |  | |  | |  | |  | 7 (54%) |
|  | United States of America | Chicago | Chicago: GO TO 2040 Regional Comprehensive Plan Chicago (2010) |  |  |  |  |  |  | |  |  | |  |  | |  | |  | |  | 5 (38%) |
|  |  |  | A Recipe for Healthy Places: Addressing the Intersection of Food and Obesity in Chicago (2013) |  |  |  |  |  |  | |  |  | |  |  | |  | |  | |  | 5 (38%) |
|  |  | New York City | Foodworks: a Vision to Improve NYCs Food System (2010) |  |  |  |  |  |  | |  |  | |  |  | |  | |  | |  | 10 (77%) |
|  |  |  | New York City Food Standards (2011) |  |  |  |  |  |  | |  |  | |  |  | |  | |  | |  | 6 (46%) |
|  |  | San Francisco | Healthy & Sustainable Food for San Francisco (2009) |  |  |  |  |  |  | |  |  | |  |  | |  | |  | |  | 6 (46%) |
|  |  |  | San Francisco Zero Waste (2018) |  |  |  |  |  |  | |  |  | |  |  | |  | |  | |  | 2 (15%) |
|  | ***Total number of policies at regional level targeting each diet-related practice*** | | | 8 | 6 | 7 | 7 | 6 | 2 | | 3 | 5 | | 6 | 5 | | 2 | | 4 | | 1 | Average 45% |
|  | ***Proportion (%) of policies targeting each diet-related practice by total number of LG food policies for region (n 10)*** | | | 80 | 60 | 70 | 70 | 60 | 20 | | 30 | 50 | | 60 | 50 | | 20 | | 40 | | 10 |  |

| *Local Government Food Policy* | | | | | *Healthy and Sustainable Diet-Related Practice^*^* | | | | | | | | | | | | | | | | | | *Diet-related practice targeted*  *n (%)* |
| --- | --- | --- | --- | --- | --- | --- | --- | --- | --- | --- | --- | --- | --- | --- | --- | --- | --- | --- | --- | --- | --- | --- | --- |
| *Global Region* | | *Country* | *Signatory City (MUFPP)* | *LG Food Policy Name* | *1* | *2* | *3* | *4* | *5* | *6* | | *7* | | *8* | *9* | *10* | | *11* | | *12* | | *13* |  |
| **Sub-Saharan Africa**  *(3 LG Food Policies)* | | Kenya | Nairobi | Nairobi Urban Agriculture Promotion and Regulation Act (2015) |  |  |  |  |  |  | |  | |  |  |  | |  | |  | |  | 3 (23%) |
|  |  | Senegal | Dakar | Dakar Micro-Gardens Programme (2006) |  |  |  |  |  |  | |  | |  |  |  | |  | |  | |  | 7 (54%) |
|  |  | South Africa | Cape Town | Urban Agriculture Policy (2007) |  |  |  |  |  |  | |  | |  |  |  | |  | |  | |  | 5 (38%) |
|  |  | **Total number of policies at regional level targeting each diet-related practice** | | | 3 | 3 | 2 | 2 | 0 | | 0 | | 0 | 2 | 1 | 2 | 0 | | 0 | | 0 | | Average 38% |
|  |  | ***Proportion (%) of policies targeting each diet-related practice by total number of LG food policies for region (n 3)*** | | | 100 | 100 | 67 | 67 | 0 | 0 | | 0 | | 67 | 33 | 67 | | 0 | | 0 | | 0 |  |
| **South Asia** | Nil | | Nil | Nil |  |  |  |  |  |  | |  | |  |  |  | |  | |  | |  | 0 |
| **East Asia & Pacific** | Nil | | Nil | Nil |  |  |  |  |  |  | |  | |  |  |  | |  | |  | |  | 0 |
| **Middle East & North Africa** | | Nil | Nil | Nil |  |  |  |  |  |  | |  | |  |  |  | |  | |  | |  | 0 |
| **Healthy and sustainable diet-related practices^[[4]](#footnote-4)^ (n 13 across three categories):*  *GREEN – ‘Where to source food’ (1) Select food grown using sustainable food production practices, valuing Indigenous knowledges, (2) Strengthen local food systems by connecting with primary producers, (3) Eat seasonally, incorporating native and wild-harvested foods, (4) Eat locally available foods,*  *BLUE – ‘What to eat’ (5) Avoid over-consumption beyond caloric requirement, (6) Consume no more than recommended amounts of animal-derived foods, (7) Limit intake of highly processed, nutrient poor and over-packaged foods, (8) Increase intake of plant-based foods, (9) Eat a wide variety of foods to promote biodiversity,*  *PURPLE – ‘How to eat’ (10) Adopt food waste-minimisation strategies, (11) Preference home-made meals and share with others, (12) Consume safe tap water as preferred drink, (13) Breastfeed infants where possible.* | | | | | | | | | | | | | | | | | | | | | | | |

**Supplementary Material D**

Names of relevant policies *referred to* within each LG food policy^[[5]](#footnote-5)^, organised by global region, country and signatory city

| *Global Region* | *Country* | *Signatory City (MUFPP)* | *LG Food Policy Name* | *Related policies referred to within the LG food policy document* |
| --- | --- | --- | --- | --- |
| **East Asia & Pacific** | Nil | Nil | Nil | Nil |
| **Europe & Central Asia** | Austria | Vienna | OkoKauf Wien ('EcoBuy') Green Public Procurement program (2012) | The ‘OkoKauf Wien’ (‘EcoBuy’) programme, with targets for organic food procurement, was launched as part of the Vienna Climate Protection Programme (KliP Wien) in 1999. [City of Vienna website](https://www.wien.gv.at/english/environment/protection/oekokauf/index.html). Animal Welfare Act (Bundestierschutzgesetz) Federal Law Gazette I No 118/2004, 1996 Regulation on Packaging, Federal Law Gazette (BGBl.) II No 648/1996, Federal Ministry of Health (trans fatty acid regulation), Federal Law Gazette II No 267/2009, Austrian Federal Procurement Act of 2006 (Bundesvergabegesetz, BVergG) |
|  | Belgium | Gent | Gent Local Food Policy "Ghent en Garde" (2016) | Food Smart Cities for Development Project (EU), Sustainable Development Goals, Milan Urban Food Policy Pact |
|  | Denmark | Copenhagen | Organic Programme - Copenhagen House of Food (2013) | Organic food in public procurement has been on the political agenda in Denmark since the 1990s as part of their sustainable food strategy. Since 2009 the Organic Programme has been administered by 'Copenhagen House of Food'. "Eco-Metropolis: Our Vision for Copenhagen 2015" states that 'there will be at least 20% organic food in the city's food consumption, with the city taking the lead with at least 90% organic food in municipal institutions'. |
|  | France | Paris | Topager "WildRoof" (2013) | The intervention received funding from the City of Paris as part of their call for project "Innovative Vegetation" for terracotta walls (Wild On Wall). The [City of Paris' food strategy](https://www.api-site.paris.fr/paris/public/2018%2F9%2FENG_Abrege_StratAlim.pdf) aims to increase urban agriculture. |
|  |  |  | Paris Strategy for Sustainable Food (2018) | 2010 Sustainable Food Plan, 2003 Green Verte Charter, Milan Urban Food Policy Pact (signed in 2015) |
|  |  |  | Main Verte ‘Green Hand’ Charter (2003) | Part of the Carrot City directory for 'Designing Urban Agriculture' in many cities globally and the [summarised charter](https://translate.google.com/translate?hl=en&sl=fr&u=https://api-site-cdn.paris.fr/images/123236.pdf&prev=search) agreement itself |

| *Global Region* | *Country* | *Signatory City (MUFPP)* | *LG Food Policy Name* | *Related policies referred to within the LG food policy document* |
| --- | --- | --- | --- | --- |
| **Europe & Central Asia** | Italy | Multiple | National Guidelines for School Catering, Italy (2010) Ancona, Bari, Bologna, Cagliari, Milan, Rome | European commission School Food Policy Country [Factsheet](https://ec.europa.eu/jrc/sites/jrcsh/files/jrc-school-food-policy-factsheet-italy_en.pdf) and [Official Policy](https://translate.googleusercontent.com/translate_c?depth=1&hl=en&prev=search&rurl=translate.google.com&sl=it&sp=nmt4&u=http://www.salute.gov.it/imgs/C_17_pubblicazioni_1248_allegato.pdf&usg=ALkJrhgda9Ulu8mr1HsBW0NUWo6xGN20QA). United Nations Decade of Action on Nutrition, UN's School Food and Nutrition Framework |
|  |  | Bologna | Community rooftop garden of Via Gandusio (2011) | Not stated however was designed by the Municipality of Bologna so must be considered part of a funded workplan. |
|  |  | Catania | ‘Orti e arte’ - Vegetable Gardens and Arts (2018) | This urban garden is part of the [EU's over-arching policy intervention](https://eur-lex.europa.eu/legal-content/EN/ALL/?uri=CELEX%3A52016IR3170) to promote urban agriculture which has since informed the development of iPES Food's "[Towards a Common Food Policy for the European Union](https://www.askfood.eu/tools/forecast/wp-content/uploads/2019/07/CFP_FullReport.pdf)" |
|  |  | Rome | Sustainable Food Procurement for Schools in Rome (2013) | ‘All for Quality' program has been in place in Rome since 2001. 'Finance Law 488' is National legislation introduced in 1999 to introduce organic procurement in school canteens. In 2010, Rome's council introduced a green procurement policy for food and canteens. [Geographical Indications and Designations of Origin EU Legislation](https://eur-lex.europa.eu/LexUriServ/LexUriServ.do?uri=OJ:L:2007:189:0001:0023:EN:PDF) & [European Parliament resolution on Fair Trade and Development](https://eur-lex.europa.eu/LexUriServ/LexUriServ.do?uri=OJ:L:2007:189:0001:0023:EN:PDF) |
|  |  | Turin | City of Turin: Guidelines on school canteen services (2015) | The evaluation results of the initial policy informed these guidelines which were to be adopted as an official political act and used in the 2018 procurement tender process. Part of the [INNOCAT project](https://sustainable-catering.eu/actions/innocat-tenders/procurement-of-eco-innovative-school-catering-services/) that aimed to bring together public/private buyers to make tenders publicly available to promote eco-innovative catering, services and solutions. |
|  | Netherlands | Rotterdam | Edible City Solution initiatives (2018) | Part of the EU-wide [Edible Cities Network](https://www.edicitnet.com/), a European demonstration program recognising the importance of urban agricultural initiatives for the city. The City of Rotterdam reports over 200 'green' initiatives and has a dedicated local government work package titled "[SuperbFood](https://edepot.wur.nl/443120) - sustainable urban and peri-urban food provision" |
|  | Spain | Barcelona | Seasonal food for kindergartens (2013) | Barcelona + Sustainable City Council Programme, adopted in 1995. This led to an intensive participatory process to develop the 'Citizen Commitment to Sustainability' for 2002-2012, which was updated to continue for 2012-2022. Greening of Council Service (2001) led to the Green Office Guide and subsequently the "+ Sustainable City Council Programme in 2006 which aims to encompass all local authority action. Adoption of four governmental regulatory measures related to contracts and procurement. |
|  |  |  | Barcelona green infrastructure and biodiversity plan (2020) | Related policy is the "The Municipal Plan of Urban Gaps with Territorial and Social Involvement ([BUITS Plan](https://ajuntament.barcelona.cat/ecologiaurbana/ca/pla-buits))" |

| *Global Region* | *Country* | *Signatory City (MUFPP)* | *LG Food Policy Name* | *Related policies referred to within the LG food policy document* |
| --- | --- | --- | --- | --- |
| **Europe & Central Asia** | Switzerland | Zurich | Sustainable Food Procurement Regulations in Zurich City (2010) | 2014 [Case Study Report](http://supurbfood.eu/scripts/document.php?id=126) of SupurbFood (Sustainable Urban and Peri-urban Food Provision) project in Zurich as an overarching project, with two related regulatory policies as mentioned in the included study. This report describes the broader policy context, EU Food labelling and Country of Origin legislation, Zurich Agricultural Act, Zurich Waste Management Act, Zurich Water Conservation Act, Zurich Environmental Protection Act, Zurich Energy Act, Zurich Spatial Planning Act, Zurich Cultural Land Initiative, Quality Charter for Agriculture and Food Economy |
|  | UK | Bristol | Bristol's Good Food Action Plan (2015) | In 2011 a Food Policy Council was formed by local government in Bristol. In 2012 the 'Good Food Charter' was produced as a "call to arms for all relevant stakeholders to adopt the principle of Good Food encompassing seven hallmarks; Good Food is not only tasty, healthy, affordable, but must also be produced and distributed in a way that it is good for nature, good for workers, good for animal welfare and good for local businesses". In 2013, the Good Food Plan for Bristol was launched. |
|  |  | Brighton & Hove | Spade to spoon: digging deeper. A food strategy and action plan for Brighton and Hove (2012) | This is the second local food strategy, this building upon success from the 2006 strategy. Evaluation of the 2006 strategy revealed that 90% of planned activities were implemented. Policies cited in the strategy include: 'Brighton & Hove City Council Domestic Waste Strategy (2009)', 'Best Food Forward, A One Planet Framework for Brighton & Hove, adopted by the City Sustainability Partnership (2011)', 'Climate Change Action Plan (2012)', 'NHS Brighton & Hove Public Health Directorate (2011)', 'National Childhood Measurement Programme (2010)' |
|  |  | London | Flagship Food Boroughs in London (2014) | [National School Food Plan 2013](http://www.schoolfoodplan.com/) which sets out 17 actions to transform what children eat in school and how they learn about food. Food Growing Schools, [Healthy Schools London](https://www.london.gov.uk/what-we-do/health/healthy-schools-london/awards/) and [London Healthy Workplace Charter](https://www.london.gov.uk/what-we-do/health/london-healthy-workplace-award/about-london-healthy-workplace-award). |
|  |  |  | The London Food Strategy (2018) | This policy document clearly states the relevant Mayoral strategies which are linked to the Food Strategy: Healthy and Sustainable Food for London: the mayor's food strategy (2008), New London Plan, the London Health Inequalities Strategy, the Mayor’s Economic Development Strategy, the London Environment Strategy, the Mayor’s Transport Strategy, Culture for All Londoners Strategy, the Mayor’s Skills for Londoners Strategy, the Mayor’s Vision for a Diverse and Inclusive City, a Tourism Vision for London, and a Vision for London as a 24-Hour City |

| *Global Region* | *Country* | *Signatory City (MUFPP)* | *LG Food Policy Name* | *Related policies referred to within the LG food policy document* |
| --- | --- | --- | --- | --- |
| **Latin America & Caribbean** | Brazil | Multiple | National School Feeding Program of Brazil - Sustainable Schools initiative (2009) Araraquara, Belo Horizonte, Curitiba, Porto Alegre, Praia, Rio de Janeiro, Sao Paulo | Brazil-FAO International Cooperation was signed in 2008 between the Federal Government of Brazil and the FAO. Aims to promote formulation and implementation of sustainable school feeding programs ('Sustainable Schools' initiative) based on the experience in Brazil. In 2009 the Family Farming Law (Law n. 11,947/2009) was accepted which requires at least 30% of funds granted by Brazilian government be used to buy food directly from family farmers. |
|  |  | Belo Horizonte | Belo Horizonte food security policy (2011) | Belo Horizonte Municipal Law No. 6.352, 15/7/1993 - established a historical milestone by creating the 'Municipal Secretariat of Supply' (organisational framework committed to food sovereignty). Municipal Law 10,255 of 13/9/2011 which establishes policy to support urban agriculture as part of the food security policy. . |
|  | Columbia | Medellin | Public Policy on Food Security, Food Sovereignty and Nutrition (2005) | The City of Medellin has had a public policy for food and nutrition security since 2005. This is part of the City Region Food System of Medellin (includes 31 municipalities). Plan for Food and Nutrition Security (SAN) for the period 2016-2028, aiming to ensure in the next 12 years a hunger-free and food-sovereign city. |
|  | Ecuador | Quito | Participatory Urban Agriculture Project (AGRUPAR) (2000) | Constitution of Ecuador of 2008, Organic Agriculture Law of 2007, Food Sovereignty Law of 2009, National Plan for Good Living of 2013, Plan of Development for the Metropolitan District of Quito of 2015, Quito’s Resilience Strategy, Quito’s Climate Action Plan, Municipal ordinance No. 084 on Social Responsibility, Municipal ordinance No. 048 on Urban Fauna. In 2012, a Resolution conferred more formality to the execution of AGRUPAR within CONQUITO, meaning that urban agriculture in Quito is now institutionalized as a permanent service. |
| **Middle East & North Africa** | Nil | Nil | Nil | Nil |
| **North America** | Canada | Toronto | Toronto Food Strategy (2010) | Adopted by the [Board of Health on 1 June, 2010](http://app.toronto.ca/tmmis/viewAgendaItemHistory.do?item=2010.HL31.1). Provincial 'Local Food Act 2013' which aims to 'foster successful and resilient local food economies and systems throughout Ontario'. Toronto Food Policy Council (formed 1990), Toronto Food Charter (2001), "Cultivating Food Connections: Toward a Healthy and Sustainable Food System for Toronto" (2010), GrowTO: An Urban Agriculture Action Plan for Toronto (2012), Toronto Agriculture Program (2013), Toronto Food Strategy has ongoing amendments to align with Toronto Public Health Plans |
| *Global Region* | *Country* | *Signatory City (MUFPP)* | *LG Food Policy Name* | *Related policies referred to within the LG food policy document* |
| **North America** | Canada | Toronto | Toronto Golden Horseshoe Food and Farming Action Plan (2012) | All 7 LGAs adopted this overarching action plan as their own local policy to streamline development, reporting, etc. Each municipality contributes funding ($30k CDN per annum) to employ the Executive Director and the part-funding of projects. Rooted in the 2005 'Greater Toronto Area Agricultural Action Plan’, applied to the City of Toronto and surrounding areas. |
|  |  |  | Toronto's Long-Term Waste Management Strategy (2016) | This food waste strategy is part of a broader ['Long Term Waste Reduction Strategy'](https://www.toronto.ca/services-payments/recycling-organics-garbage/long-term-waste-strategy/waste-reduction/) ([summary document](https://www.hdrinc.com/sites/default/files/2017-06/toronto-long-term-waste-management-strategy.pdf)). [Love Food Hate Waste Canada](https://lovefoodhatewaste.ca/), 'Waste Free Ontario Act'. Love Food Hate Waste Canada is a Nation-wide campaign, facilitated at local government level. The Long-Term Waste Management Strategy was endorsed by Toronto City Council in 2016 with the intention to guide policy decisions for 30-50 years. |
|  |  | Vancouver | Regional Food System Action Plan Metro Vancouver (2016) | [Regional food system strategy Metro Vancouver](http://www.metrovancouver.org/services/regional-planning/PlanningPublications/RegionalFoodSystemStrategy.pdf) (2011) This strategy is linked to a number of existing plans e.g. Regional Growth Strategy, Integrated Solid Waste and Resource Management Plan, Affordable Housing Strategy, Drinking Water Management Plan, Parks and Greenways Plan. The strategy describes its' alignment with four key provincial initiatives; 'BC Agricultural Plan: Growing a healthy future for BC families (2008)', 'BC Climate Action Plan (2008)', 'Living Water Smart: BC's Water Plan (2008)', 'Healthy Eating Strategy (2007)' |
|  | United States of America | Chicago | Chicago: GO TO 2040 Regional Comprehensive Plan Chicago (2010) | Promote sustainable local food' is one of the 12 recommendations included in the GO TO 2040 Regional Comprehensive Plan Chicago. The plan has four themes, and this food-related recommendation fits within the 'Liveable Communities' theme. The Federally funded 'Farm Bill', legislation passed every five years to guide national agriculture policy. This plan acknowledges a shift in federal policy towards supporting local food, with modest increase in the 2008 Farm Bill for production and access to local food (e.g. Farmers' Market Promotion Program or USDA "Food Desert" study) |
|  |  |  | A Recipe for Healthy Places: Addressing the Intersection of Food and Obesity in Chicago (2013) | City of Chicago. A Recipe for Healthy Places: Addressing the Intersection of Food and Obesity in Chicago; City of Chicago Department of Housing and Economic Development: Chicago, IL, USA, 2013. |
|  |  | New York City | Foodworks: a vision to improve NYCs food system (2010) | PlaNYC is a blueprint for city-wide sustainability published in 2007, however food was missing from the narrative. Less than 3 years later, Foodworks NYC Report was released (a food sustainability blueprint). FRESH program, Green Cart program, Supplemental Nutrition Assistance Program (SNAP), Child Nutrition Act, WIC program, SNAP Education |
| *Global Region* | *Country* | *Signatory City (MUFPP)* | *LG Food Policy Name* | *Related policies referred to within the LG food policy document* |
| **North America** | United States of America | New York City | New York City Food Standards (2011) | [Supporting documentation](https://www1.nyc.gov/site/doh/health/health-topics/healthy-workplaces.page), Mandatory standards for City Facilities and Vendors: 'Meals/Snacks Purchased and Served Standards', 'Meetings and Events Standards', 'Beverage Vending Machines Standards', 'Food Vending Machines Standards', 'Commissaries Standards'. Voluntary Adoption of the Standards: 'Cafeterias/Cafes Standards |
|  |  | San Francisco | Healthy & Sustainable Food for San Francisco (2009) | Foodshed Assessment was published in 2008, the Mayor called for the Healthy and Sustainable Food for San Francisco in 2009, the Food Policy Council was formed and implemented the action items. [Think globally – eat locally](https://4aa2dc132bb150caf1aa-7bb737f4349b47aa42dce777a72d5264.ssl.cf5.rackcdn.com/ThinkGloballyEatLocally-FinalReport8-23-08.pdf): San Francisco Foodshed Assessment' (2008) American Farmland Trust, Sustainable Agriculture Education and University of California Berkeley. Executive Directive 10-01 ['Healthy Food & Beverage Options in Vending Machines'](https://sfgov.org/sffood/sites/default/files/Documents/sffood/may_2010/vending_maching_policy.pdf) (2010) |
|  |  |  | San Francisco Zero Waste (2018) | [Resolution Setting Zero Waste Date 2003](https://sfenvironment.org/sites/default/files/editor-uploads/zero_waste/pdf/resolutionzerowastedate.pdf), C40 Cities Advancing Towards Zero Waste [Declaration](https://www.c40.org/other/zero-waste-declaration), Mandatory Recycling and Composting [Ordinance](https://sfenvironment.org/sites/default/files/editor-uploads/zero_waste/pdf/resolutionzerowastedate.pdf) 2009, Single-use Food Ware Plastics, Toxics and Litter Reduction [Ordinance](https://sfbos.org/sites/default/files/o0294-18.pdf) 2018, Food Service Waste Reduction [Ordinance](https://sfenvironment.org/sites/default/files/editor-uploads/zero_waste/pdf/sfe_zw_food_service_waste_reduction_ordinance.pdf) 2006, Mayor's [Executive Order](https://sfenvironment.org/sites/default/files/editor-uploads/zero_waste/pdf/sfe_zw_executive_order_bottled_water.pdf.pdf) on Bottled Water 2007 |
| **South Asia** | Nil | Nil | Nil | Nil |
| **Sub-Saharan Africa** | Kenya | Nairobi | Nairobi Urban Agriculture Promotion and Regulation Act (2015) | Harare Declaration on Urban and Peri-urban Agriculture (2003) - endorsement from Ministers of Kenya, Malawi, Swaziland, Tanzania and Zimbabwe to develop policies and instruments to enable urban agriculture. This catalysed development of the 2015 Act, a U-turn in policy after a time when local governments were reprimanding citizens for attempting to produce food on open land, believing it to be a threat to public health and land rights. Referred to in the Act: National Agriculture, Fisheries & Food Act (2013), Crops Act (2013) |
|  | Senegal | Dakar | Dakar Micro-gardens Programme (2006) | Part of the 2019-2023 Senegalese Country Programming Framework (CPP) - in partnership with FAO. |
|  | South Africa | Cape Town | Urban Agriculture Policy (2007) | Integrated Environment Management Plan, Economic Development Strategy, Poverty Alleviation Strategy, Water Services Development Plan |

1. Tricco, A.C., et al., PRISMA Extension for Scoping Reviews (PRISMA-ScR): Checklist and Explanation*.* Ann Intern Med. 2018. 169(7): p. 467-473. [↑](#footnote-ref-1)
2. Barbour, L., et al., Local urban government policies to facilitate healthy and environmentally sustainable diet-related practices: A scoping review*.* Public Health Nutrition. 2021: p. 1-36. [↑](#footnote-ref-2)
3. Barbour, L., et al., Local urban government policies to facilitate healthy and environmentally sustainable diet-related practices: A scoping review*.* Public Health Nutrition. 2021: p. 1-36. [↑](#footnote-ref-3)
4. Barbour, L.R., Woods, J.L. and Brimblecombe, J.K. (2021), Translating evidence into policy action: which diet-related practices are essential to achieve healthy and sustainable food system transformation?. Australian and New Zealand Journal of Public Health, 45: 83-84. <https://doi.org/10.1111/1753-6405.13050> [↑](#footnote-ref-4)
5. City-level policies analysed are those identified through a scoping review of the peer-reviewed literature: Barbour L, Lindberg R, Woods J, Charlton K, Brimblecombe J. Local urban government policies to facilitate healthy and environmentally sustainable diet-related practices: A scoping review. Public Health Nutr. 2021 Oct 25:1-36. doi: 10.1017/S1368980021004432. Epub ahead of print. PMID: 34693899. [↑](#footnote-ref-5)
